# Second primary cancers in patients with gastric cancer

**DOI:** 10.2478/v10019-010-0048-2

**Published:** 2010-11-16

**Authors:** Oktay Buyukasik, Ahmet Oguz Hasdemir, Yusuf Gulnerman, Cavit Col, Ozgur Ikiz

**Affiliations:** 1 Department of General Surgery, Medical Faculty of Abant Izzet Baysal University, Bolu, Turkey; 2 Department of General Surgery, Ministry of Health Yildirim Beyazit Research and Training Hospital, Ankara, Turkey; 3 Department of General Surgery, Ministry of Health Research and Training Hospital, Adana, Turkey

**Keywords:** gastric cancer, second primary cancers, synchronous cancers, metachronous cancers

## Abstract

**Background:**

The risk of developing a second primary tumour in patients with gastric carcinoma is higher than among the general population. The aim was to investigate the clinicopathological characteristics of the second primary cancers in patients with gastric cancer in this study.

**Patients and methods:**

In the retrospective study, patients with gastric cancers were evaluated between 1995 and 2005 for primary tumours according to Warren and Gates’ criteria related with the second primary cancers.

**Results:**

Nine of the 112 patients with gastric cancer had second primary cancers. Seven of the patients were males and two females. Six patients with gastric cancers had synchronous, and three had metachronous tumours. The age of the patients ranged from 53 to 78 years, and the mean age was 61 ± 8.3 years. The most frequent site of occurrence of the second tumours was the colo-rectum (33%) followed by the upper respiratory system (22%), and the urogenital system (22%) in descending order of frequency.

**Conclusions:**

The incidence of the second primary cancer in gastric cancer patients was 8% in the current report. It is recommended that careful preoperative and postoperative examinations for other primary cancers, as well as for the extent of the primary gastric carcinoma, are carried out. Because colorectal cancer was the most common carcinoma combined with gastric carcinoma, the surveillance for this carcinoma (e.g., colonoscopy, abdominopelvic CT) would be appropriate after the diagnosis of gastric carcinoma.

## Introduction

The first description of the term “multiple primary neoplasm” by Billroth in 1889 is defined as the development of more than one neoplasm in a patient.^1^ Until the study of 1,259 case reports by Warren and Gates in 1932, multiple primary neoplasia were not taken seriously.^2^ The increase of the average life span due to the improvement in the standard of living, the increase of exposure to carcinogens, and the positive progress in cancer prognosis result in a rise in the initiation of the second primary cancers (SPC).^3^,^4^

Worldwide, gastric cancer is the fourth most common cancer in population.^5^,^6^ Nearly 70% of new cases is reported in developing countries. Gastric cancer is the second leading cause of cancer death in men and the fourth among women.^7^ In Turkey, gastric cancer is the fifth most common cancer with an incidence of 10 per 100 000.^8^ Recently, with new screening programs and the increase in the use of endoscopy, the early diagnosis of gastric cancer has increased and the survival has been prolonged with gastrectomy and extended lymph node dissections.^9^–^13^

The risk of development of a SPC in patients with cancer is increased considerably when compared to the general population.^3^ Genetic, environmental and other (chemotherapy, radiotherapy etc.) factors are held responsible for the development of synchronous or metachronous (pre, post) SPC in patients with gastric cancer.

In this retrospective study, the aim was to investigate the frequency and clinicopathological characteristics of SPCs associated with gastric cancers.

## Patients and methods

In this study, 112 patients who underwent the surgical treatment consecutively between 1995 and 2005 were evaluated retrospectively. Gastric cancer was diagnosed and confirmed by endoscopic biopsy in all patients. The patients were investigated through thoraco-abdominal imaging (CT and ultrasonography) following the routine examinations and clinical TNM staging was conducted. Clinical properties, operative findings and pathological results were evaluated from hospital records. The study were excluded those patients who were not operated on for gastric cancer due to the inoperability of their advanced gastric cancer stage.

SPC was evaluated according to Warren and Gates criteria, requiring that both tumours should be histologically malignant. There should be minimum 2 cm of tumour free tissue between the two lesions; the duration between diagnoses should be minimum 5 years if the tumours are in the same localization; and it should be demonstrated that the second tumour is not metastasis.^2^ SPC is called a synchronous tumour if diagnosed at the same time with the first cancer or within the following six months, while it is called metachronous tumour if diagnosed later than six months.

## Results

SPC was observed in nine of 112 patients with gastric cancer (8%). Six of these patients had synchronous cancer, while the remaining three had metachronous cancer. Seven of the patients were males and two females, and the mean age was 61.0 ± 8.3 years. According to the income, the patients had a lowest socioeconomic level and seven of them were consumed tobacco more than 20 pack-years. No data concerning the presence of malignant diseases was found in the family histories of the patients.

Six patients had *synchronous* SPC. In one patient, during the endoscopic retrograde cholangiopan-creatography (ERCP) performed while aetiology of extra hepatic cholestasis was being investigated, both antrum and ampulla Vater carcinomas were observed. As the second patient was being examined for complaints such as dysphonia and dysphagia, cancer of the larynx accompanying gastric cancer was observed. During the abdominal exploration, cancer of the appendix was observed in one of the patients who underwent the surgical treatment after the diagnosis of gastric cancer, while in another patient cancer of the colon was found. In a patient with gastric cancer, in the abdominal tomography performed for the preoperative evaluation, tumours were observed on both kidneys and the postoperative histopathological diagnosis was reported as renal cell cancer. The last patient in the synchronous tumour group was operated for cancer of the colon six months previously, and gastric cancer was found in the upper gastrointestinal system; endoscopy was performed during the postoperative follow-up period.

There were three patients with *metachronous* SPC: One patient had been followed in the urology department for urinary bladder cancer for three years. During laparotomy, it was found that local and at advanced stage (Stage IV). The second patient had gone through “modified radical mastectomy” due to breast cancer. In the third year of the follow up period, gastric cancer was observed. The third patient had undergone total laryngectomy and the radical cervical dissection because of cancer of the larynx five years previously.

The clinical characteristics of patients, the treatment methods and survivals are summarized in Table 1. Gastric cancer was found in three patients in the oncological follow up period. One patient had gone through right hemicolectomy for cancer of the colon six months previously. In the other five patients, the cancer was observed in the investigations done during the second malignity preoperative investigation period or in routine abdominal explorations done during the surgical treatment.

## Discussion

The common use of further diagnostic methods has increased the rate of the early diagnosis of malignancy.^14^ Due to the early diagnosis of cancers and current treatment methods the survival in patients with cancer has gone up recently when compared to previous years.^4^ Compared with the general population, cancer patients were at a nearly increased risk for new primary cancer after cancers at many sites.^3^

In the presence of multiple cancers, there has been no discussion on the patients’ prognosis being worse. However, although there have been plenty of clinical studies about such patients, the number of studies about treatment methods and how the prognosis is influenced has been quite limited. In patients with multiple cancers, cancer can be detected in different organs and their stagings are not homogenous. Furthermore, the patients cannot be classified in groups and comparative results cannot be deduced due to different methods of the treatment.

It is well known that synchronous and/or metachronous cancers can be observed in the same or different organs. These include oesophageal with oropharyngeal cancer or gastric carcinoma, breast carcinoma with contralateral breast carcinoma or endometrial carcinoma, colon and rectal carcinoma with another colorectal primary or genitourinary primary tumour, and multiple primary cancers within the urinary tract. It is thought that the presence of cancer in the family history, genetic factors and chemotherapy and radiotherapy applications affect the formation of multiple cancers. Despite the fact that there have been studies showing that the synergy of gastric cancer and other cancers can be due to certain disorders, such as microsatellite instability, germ-line mutations and E-cadherin, TP53, RAS mutation; the molecular basis of the formation of tumours is still not understood completely.^15^–^17^

The frequency of SPC-associated gastric cancer was found as 8% in the current study. As it can be seen in Table 2, in previous studies this rate varies between 1 and 4.2%.^9^,^10^,^18^–^23^ Our incidence was higher than that of other reports. In this study, the number of patients with second primary cancers is small and thus important statistical limitations exist. A better improved national cancer record system and broader series are required. However, this higher incidence might be due to the fact that this study analyzed patients who had undergone surgical intervention. On the other hand, in order to draw attention to the fact that the frequency of SPC in Turkey is higher than other societies

Concerning second primary cancers in gastric cancer patients, environmental factors, such as dietary habits or tobacco use, and genetic factors have also been suggested to be risk factors. In published series, it is seen that the frequency of finding SPC accompanying gastric cancer is higher in older male patients, as it is in other types of cancer.^3^,^10^,^18^,^22^ In the series presented, the mean age was 61.0 ± 8.3 (Min 53 - Max 78), the male/female ratio was 7/2. It is accepted that smoking is an important risk factor for multiple primary cancers just as in other types of cancers. In this study, it was observed that 78% of the patients consumed tobacco.

The tumour determining the prognosis in SPC is gastric cancer.^16^ However, second malignancies in patients with early gastric cancer have a determinant effect on prognosis.^22^ The method of the treatment of synchronous cancers should include the primary cancer about which malignancy prognosis is expected. Synchronous cancer should be treated with curative intent if possible.^5^

In a study in which gastric cancer was investigated as the SPC, an increase in gastric cancer development risk was observed within 10 years following cervical, ovarian, testicular cancers and Hodgkin and non-Hodgkin lymphoma. Treatments of the initial cancer, such as the radiation therapy or chemotherapy were found to be responsible for this increase in risk in gastric cancer and SPC.^24^ It is stated that the highest risk is after esophagus cancer in sporadic patients.^5^ In the patients followed in the current study, gastric cancer developed as SPC following breast, larynx and bladder cancer. These are sporadic cases.

Hereditary conditions known to increase the risk of gastric cancer include: familial cancer syndromes such as hereditary non-polyposis colorectal cancer, Li-Fraumeni syndrome, breast-ovarian cancer syndrome, multiple endocrine neoplasia Peutz-Jegher syndrome and Cowden syndrome.^2^ In hereditary cancers, the average age of onset for the gastric cancer is in the late 30s with the majority of cancers occurring before age 40.

Among the SPCs associated with gastric cancer, colorectal cancers are the most frequent and their rate is reported to be between 20% and 70%.^19^,^21^ In our study, synchronous colorectal cancer was found (33.3%) in three patients (of the colon in two patients and of the appendix in one patient). It is reported that lung cancer is the second most frequently occurring cancer among all SPCs.^3^,^9^,^18^,^19^,^21^ Many researchers state that performing gastroduodenoscopy and colonoscopy is necessary in the preoperative evaluation and in the postoperative follow up of patients with gastric and colorectal cancers; whereas some researchers suggest that bronchoscopy should be added to these investigations.^14^,^21^,^25^

In the present series, the rate of SPC associated with gastric cancer is 8%. This relatively high rate indicates that it would be highly significant to pay attention to the development of SPC in the preoperative evaluation period, in the exploration during surgery, and in the postoperative follow up period. The systemic investigation and examination of patients with gastric cancer should be performed in detail, and all organs should be examined carefully during surgery. In addition, while investigating possible recurrence and metastasis of the primary cancer, all systems should be examined for the possible presence of SPC. Not only for patients with gastric cancer, but also for all malignant cases it is extremely necessary to inform the patients about signs and symptoms of other organs and to perform the systemic investigation and the full physical examination in each control in case multiple primary cancers develop. The early diagnosis of SPC provides a longer survival and a better quality of life.

To conclude, the probability of the development of multiple primary cancers should be considered in the diagnosis and the treatment of all malignant tumours. Since gastric and colorectal cancer synergy is quite frequent, the importance of gastrointestinal panendoscopy should be highlighted. The treatment should be appropriate for observed synchronous or metachronous cancer and it is necessary to try to be treated with curative intent in combined radical resections.

## Figures and Tables

**TABLE 1.** Clinicopathological characteristics of patients, applied treatment methods and survivals

**Age/Sex**	**Primary Cancer**			**Secondary primary cancer**					**Operation/Procedure**	**Adjuvant Chemotherapy**	**Survivals**	**Cause of death**

	**Organ**	**Pathology**	**TNM**	**Organ**	**Time**	**Location**	**Pathology**	**TNM**				
77/M	**Stomach**	Adeno-Ca	T3N0M0	Colon	***Synchronous***	Ascending colon	Adeno-Ca	T2N0M0	Distal esophagectomy + Proximal gastrectomy + Splenectomy + D2 Lymph dissection + Right hemicolectomy	6 courses of 5- FU + Ca Folinat	Disease free for 9 years	Alive
58/M	**Stomach**	Adeno-Ca	T4N1M0	Kidney	***Synchronous***	Both kidneys	Renal Ca (Clear cell ca)	T2N0M0	Distal subtotal gastrectomy + D2 Lymph dissection + Bilateral partial nephrectomy	-	22 months	Liver and pulmoner metastases (Renal cancer)
78/M	**Stomach**	Adeno-Ca	T2N1M0	Appendix	***Synchronous***	Appendix	Mucinous carcinoma	T3N0M0	Distal subtotal gastrectomy + D2 Lymph dissection + Appendectomy	6 courses of Etoposide + Doxorubicine + Cisplatin	Disease free for 43 months	Alive
68/M	**Stomach**	Adeno-Ca	T3N0M0	Larynx	***Synchronous***	Larynx	Squamous Cell Ca	T3N1M0	Total gastrectomy + D2 Lymph dissection + Laryngectomy + Bilateral neck dissection	Radyotherapy (Neck) 6 courses of Cisplatin + 5-FU	Disease free for 15 months	Alive
54/F	**Stomach**	Adeno-Ca	T2N0M0	Pancreas	***Synchronous***	Ampulla Wateri	Adeno-Ca	T2N0M0	Whipple procedure	6 courses of 5-FU + Ca folinat	Disease free for 45 months	Alive
50/F	Colon	Adeno-Ca	T4N1MO	**Stomach**	***Synchronous*** (6 months ago)	Stomach	Adeno-Ca	T3N1M0	Distal subtotal gastrectomy + D2 Lymph dissection	6 courses of 5-FU + Ca folinat + 3 courses of Etoposide + Doxorubicine + Cisplatin	18 months	Brain, liver and pulmoner metastases
63/M	Bladder	Transitional cell-Ca	T2N0M0	**Stomach**	3 years ago	Stomach	Adeno-Ca	T4N1M1	Laparotomy	?	4 months	Peritonitis carcinomatosa
53/M	Larynx	Squamosus cell-Ca	T2N0M0	**Stomach**	5 years ago	Stomach	Adeno-Ca	T4NIM0	Distal subtotal gastrectomy + D2 Lymph dissection	6 courses of 5- FU + Ca Folinat	12 months	Peritonitis carcinomatosa and liver metastasis
67/F	Breast	Invasive ductal-Ca	T2N1M0	**Stomach**	3 years ago	Stomach	Adeno-Ca	T3N1M0	Distal subtotal gastrectomy + D2 Lymph dissection	6 courses of CMF + Tamoxifen	4 months	Cerebral embolisation

**TABLE 2.** The rate of gastric cancer and secondary primary cancer

	**Büyükaşık O (2010)**	**Ikeda Y (2003)**	**Lee HJ (2006)**	**Park YK (2005)**	**Eom BW (2008)**	**Dinis RM (2002)**	**Muela M (2006)**	**Ha TK (2007)**
Number of patients with gastric cancer (n)	112	2250	3291	2509	4593	2668	1170	10090
Secondary primary cancer n (%)	9 (8%)	95 (4.2%)	111 (3.4%)	65 (2.6%)	159 (3.4%)	78 (3.4%)	23 (1.96%)	96 (%1.0)
Synchronous tumours	6 (66%)	48 (50.5%)	111 (100%)	17 (26.2%)	49 (30.8%)	21 (27%)	12 (52%)	96 (%100)
Metachronous tumours	3 (33%)	47 (49.5%)		48 (73.8%)	110 (69.2%)	57 (73%)	11 (48%)	
Pre metachronous	3 (33%)			36 (55.4%)	42 (26.4%)			
Post metachronous	0			12 (18.4%)	68 (42.8%)			
